# How Workplace Fun Promotes Employees’ Innovative Behavior: A Dual Mediation Model

**DOI:** 10.3390/bs16050750

**Published:** 2026-05-12

**Authors:** Wenwei Wang, Jie Tang

**Affiliations:** 1 School of Education and Psychology, Minnan Normal University, Zhangzhou 363000, China; wangwenwei@mnnu.edu.cn; 2 School of Economics, Fujian Normal University, Fuzhou 350117, China

**Keywords:** fun activities, innovation, positive affect, the motivational dimensional model of affect, Affective Events Theory, Chinese culture

## Abstract

This study examines how two types of workplace fun—sociality-oriented fun and assistant-oriented fun—relate to different forms of employees’ innovative behavior through positive affect and job satisfaction, drawing on the Affective Events Theory and the motivational dimensional model of affect. Data were collected from 17 supervisors and 116 employees in a Chinese state-owned electric power company using a supervisor-subordinate dyadic survey procedure. Mplus 8.3 was used to test the hypothesized model. The results show that sociality-oriented fun is positively associated with affect-driven innovative behavior through high-motivated positive affect, whereas assistant-oriented fun is positively associated with judgment-driven innovative behavior through the serial mediating role of low-motivated positive affect and job satisfaction. These findings provide a more differentiated understanding of how workplace fun may shape employee innovation and offer practical implications for designing fun activities that align with different innovation goals.

## 1. Introduction

In the digital age, employees are increasingly required to demonstrate creativity in response to complex and dynamic competitive environments (W. Chen et al., 2024). At the same time, workplace fun has emerged as an important human resource management practice and is increasingly recognized as a key driver of employee motivation, well-being, and positive workplace experiences (Han et al., 2024; Michel et al., 2019; Tews et al., 2021). This approach is exemplified by companies such as Google, which hosts annual April Fool’s Day celebrations, encourages employees to play table tennis during breaks, and allows them to personalize their workspaces (F. R. Yang & Chen, 2023). Simultaneously, academic interest in the implications of workplace fun has been growing. Previous studies suggest that fun activities at work foster diverse facets of employee innovation, including creativity (Wu & Zhang, 2024), performance creativity (Fluegge-Woolf, 2014), engagement in the creative process (Qureshi et al., 2022), and innovative conduct (Jing et al., 2021; Lee et al., 2021). In recent years, research on workplace fun has expanded from general positive outcomes to more specific behavioral consequences and explanatory mechanisms. Existing studies suggest that workplace fun can foster creativity and innovative behavior (Jing et al., 2021; Lee et al., 2021; Qureshi et al., 2022; Wu & Zhang, 2024), thriving at work (Han et al., 2024), service performance (M. Chen et al., 2025), and lower job stress (Islam & Alam, 2026), through mechanisms such as work engagement (Qureshi et al., 2022), affective commitment (Jing et al., 2021), task crafting (J. Yang et al., 2024), and reduced burnout (Lee et al., 2021). Together, these studies help explain how a fun work environment promotes employee innovation.

Although previous studies provide growing evidence that workplace fun is positively associated with employee innovation, important theoretical questions remain unresolved. Existing research has linked workplace fun to a range of innovation-related outcomes, such as creativity, creative process engagement, and innovative behavior (J. Yang et al., 2019; Jing et al., 2021; Lee et al., 2021; Qureshi et al., 2022). However, this literature has tended to treat workplace fun as a broadly positive workplace experience and has paid less attention to whether different forms of workplace fun may operate through different psychological mechanisms.

A similar limitation appears in the innovation literature. Innovative behavior is often treated as a general outcome, yet the Affective Events Theory (AET; Weiss & Cropanzano, 1996) suggests that workplace events may influence behavior through different pathways, including more immediate affect-driven responses and more evaluative judgment-driven processes. At the same time, affective mechanisms are often discussed in broad terms, even though positive affect may differ in its motivational qualities and behavioral consequences (Gable & Harmon-Jones, 2010, 2016). Taken together, these limitations suggest that the key issue is not simply that few studies have examined workplace fun and innovation, but that limited attention has been paid to how different forms of workplace fun may influence different forms of innovative behavior through differentiated affective mechanisms. Although recent studies have begun to examine more specific mechanisms and moderators, such as autonomous motivation, task crafting, and manager support for fun (Han et al., 2024; J. Yang et al., 2024), the literature still lacks an integrated explanation of how differentiated forms of workplace fun may be linked to differentiated affective pathways and distinct forms of innovative behavior.

Accordingly, the purpose of this study is not to claim a completely unexplored relationship, but to provide a more differentiated explanation of how workplace fun may be linked to employee innovation. More specifically, we distinguish between two forms of workplace fun, two types of positive affective experience, and two forms of innovative behavior in order to examine whether different workplace fun activities may be associated with different innovation-related pathways.

Building on AET (Weiss & Cropanzano, 1996) and the motivational dimensional model of affect (Gable & Harmon-Jones, 2010, 2016), we further propose that positive affect is not a unitary mechanism. Different workplace events may evoke different forms of positive affect depending on their social meaning, spontaneity, and work relevance. In turn, these affective states may channel employees toward different innovative responses. High-motivated positive affect (HMPA) is more action-oriented and therefore more likely to facilitate affect-driven innovative behavior (ADIB), whereas low-motivated positive affect (LMPA) is more reflective and may first shape employees’ work evaluations, such as job satisfaction, before contributing to judgment-driven innovative behavior (JDIB; Weiss & Cropanzano, 1996; Gable & Harmon-Jones, 2010, 2016).

Specifically, this study focuses on two contrasting forms of workplace fun, namely sociality-oriented fun and assistant-oriented fun, and examines whether they influence different forms of innovative behavior through differentiated affective pathways. By integrating distinctions in workplace fun, positive affect, and innovative behavior, this study seeks to contribute to the workplace fun and innovation literature in a more fine-grained manner and responds to previous calls to clarify the mechanisms through which workplace fun contributes to employee innovation (Tang et al., 2017; Michel et al., 2019; T. Wang et al., 2019; J. Yang et al., 2019).

## 2. Theory and Hypotheses

### 2.1. Workplace Fun

Scholars generally agree that workplace fun refers to a range of playful activities that provide amusement, enjoyment, and pleasure in the work environment (Ford et al., 2003; Boekhorst et al., 2021). However, there is still considerable debate about its boundaries, especially regarding where and when these activities occur. Recent studies also suggest that the meaning and manifestation of workplace fun continue to evolve across organizational settings, including hybrid work arrangements, co-working spaces, and team-based contexts (Lee et al., 2022; Plester & Lloyd, 2024; Wu & Zhang, 2024). Guided by Tang et al. (2017) and Y. Wang et al. (2017), we argue in this study that the scope of workplace fun encompasses three aspects: (1) Activities initiated by organizations to make employees happy, regardless of where they occur or whether they are work-related; (2) Activities within the workplace, initiated by employees themselves, which create a sense of fun, regardless of work hours; and (3) Activities among colleagues, regardless of their occurrence within the workplace or working hours.

Furthermore, although workplace fun is recognized as a multidimensional construct, little research has examined its classification, and few empirical studies have been based on a categorized model (W. Liu et al., 2017). Existing empirical studies on multidimensional workplace fun primarily utilize either all or individual dimensions informed by the following frameworks: (1) A four-dimensional model comprising coworker socialization, celebration activities, personal freedom, and global fun (Fluegge, 2008); (2) A three-dimensional model encompassing fun activities, fun job responsibilities, and fun coworker interactions (Tews et al., 2012); (3) Another three-dimensional model consisting of fun activities, coworker socialization, and manager support (Tews et al., 2014).

However, there are three problems with studies on this topic. Firstly, dimension classifications lack sufficient theoretical basis and empirical research to test them. Secondly, the understanding and measurement of conceptual dimensions are inconsistent among scholars, leading to discrepancies and even contradictions in research findings (J. Yang et al., 2019). Finally, cultural context may influence the type and measurement of workplace fun. Considering these challenges, and taking into account the specific cultural and social context, we adopt the types of workplace fun classified by Y. Wang et al. (2017). Using the grounded theory methodology, workplace fun is categorized into four dimensions: sociality-oriented, relaxation-oriented, assistant-oriented, and welfare-oriented fun, based on the initiator of fun activities and their relevance to work. We then further select two distinct and frequently observed types of workplace fun in Chinese organizations to study their effects. The first type is sociality-oriented fun, which refers to employee-initiated activities that are less related or unrelated to work, such as jokes, humor, or online social interaction. The second type is assistant-oriented fun, which refers to organization-initiated activities that are closely related to employees’ work, such as a small party organized by a team to celebrate good performance (Y. Wang et al., 2017).

Rather than examining all four dimensions simultaneously, this study focuses on sociality-oriented fun and assistant-oriented fun for both theoretical and contextual reasons. Theoretically, these two forms represent a meaningful contrast along two dimensions central to the present model: the initiator of the activity (employee-initiated versus organization-initiated) and the degree of work relevance (less work-related versus highly work-related) (Y. Wang et al., 2017). This contrast makes them particularly suitable for examining whether different types of workplace fun evoke different affective states and ultimately different forms of innovative behavior.

Contextually, these two forms are also frequently observed in Chinese organizations and are likely to carry distinct meanings for employees. Sociality-oriented fun is more spontaneous and relational in nature, whereas assistant-oriented fun is more structured and embedded in task-related organizational practices (Y. Wang et al., 2017). Focusing on these two contrasting forms therefore allows us to develop a more targeted explanation of how workplace fun may operate through differentiated affective mechanisms.

### 2.2. Workplace Fun and Employees’ Innovative Behavior

Employees’ innovative behavior is characterized as voluntary, active, and non-organizational (Parker & Collins, 2010). AET (Weiss & Cropanzano, 1996) is commonly used to explain how work events influence employees’ innovative behavior. According to AET, there are two types of innovative behavior with different triggers: affect-driven innovative behavior (ADIB), which occurs when employees generate novel ideas due to highly activated positive affect; and judgment-driven innovative behavior (JDIB), which happens when affective reactions first influence employees’ attitudes and evaluations before leading them to exhibit certain forms of innovation.

Building on the AET, Lee et al. (2021) investigated an underlying mechanism wherein “play at work”—a concept intrinsically linked to workplace fun—influences employees’ innovative behavior by reducing experienced burnout as the mediating process. J. Yang et al. (2019) demonstrated that different aspects of workplace fun, such as group activities, social interaction, and autonomous fun at work, encourage employees’ innovative behavior along diverse paths, validating the mediating effect of experienced fun between workplace fun and employees’ innovative behavior.

In light of these findings, this paper aims to further examine the influence of two types of workplace fun on two forms of employee innovative behavior. First, whether it is initiated by the organization (assistant-oriented) or by employees (sociality-oriented), workplace fun can enhance affective connections among employees or between employees and their organization, making them feel understood and supported by others (J. Yang et al., 2019). Previous studies have found evidence that employee innovation tends to increase with organizational support (Rahmaningtyas et al., 2022) and coworker support (Bani-Melhem et al., 2018).

Furthermore, workplace fun provides an environment that encourages free expression where brainstorming occurs unconsciously, liberating individuals from fixed thinking modes and opening up new perspectives; this collision of ideas is often where innovation begins (Shi & Yao, 2019). Frequent communication promotes the sharing and integration of knowledge among colleagues, which can lead to more innovative activities (Zhang et al., 2016).

Moreover, workplace fun can increase employee enjoyment and motivation to complete work tasks (Plester & Hutchison, 2016), ultimately leading to greater creative engagement in their work (K. Liu & Ge, 2020; Qureshi et al., 2022). Recent findings also suggest that workplace fun may influence innovation through multiple pathways and under different contextual conditions, rather than through a single uniformly positive process (Wu & Zhang, 2024; J. Yang et al., 2024). Sociality-oriented fun is more likely to create a spontaneous, energized, and socially expressive atmosphere, which may be especially relevant to the generation of novel ideas and other affect-driven forms of innovation. By contrast, assistant-oriented fun is more closely embedded in work processes and may strengthen employees’ positive evaluations of their work context, thereby making it more relevant to innovation that depends on appraisal, persistence, and implementation (Weiss & Cropanzano, 1996; J. Yang et al., 2019). For this reason, distinguishing between ADIB and JDIB allows a more precise examination of how workplace fun contributes to employee innovation. Accordingly, we hypothesize that:

**H1a.**  *Sociality-oriented fun has a positive effect on ADIB*.

**H1b.**  *Sociality-oriented fun has a positive effect on JDIB*.

**H1c.**  *Assistant-oriented fun has a positive effect on ADIB*.

**H1d.**  *Assistant-oriented fun has a positive effect on JDIB*.

### 2.3. The Mediating Role of High-Motivated Positive Affect

To clarify the topic, it is important to distinguish among emotions, moods, and affect. Emotions, when contrasted with moods, are characterized by shorter durations, lesser intensity, and typically have a specific stimulus (Frijda, 1993). Although distinct from moods, previous studies have shown that positive emotions and positive moods produce similar outcomes on cognitive processes. It is difficult to distinguish between emotions and moods at the measurement level (Gable & Harmon-Jones, 2010). Affect is a broader concept often conceptualized as two dimensions: valence and arousal (Russell & Barrett, 1999). In this study, we use the term affect as the focal construct because our theoretical argument concerns broad positive affective activation rather than short-lived discrete emotions or diffuse moods. Accordingly, to maintain conceptual consistency, we use affect throughout the paper.

Positive affect reflects a sense of pleasure that arises when individuals perceive their current circumstances as favorable or promising. A body of research demonstrates that positive affect promotes individual mental health (Harker & Keltner, 2001) and physical health (Ostir et al., 2000), as well as improving organizational efficiency (Staw et al., 1994). In recent years, developmental psychology research has been delving deeper into the categorization of affect. Among these perspectives, Gable and Harmon-Jones’ motivational dimensional model of affect proposes that, in addition to valence and arousal, motivation also has direction and intensity. Positive affect can be divided into HMPA, which narrows cognition and makes individuals more persistent in pursuing goals, and LMPA, which broadens cognition, helps individuals examine environmental cues more fully, and encourages exploratory behavior (Gable & Harmon-Jones, 2016).

We argue that high-motivated positive affect (HMPA) is especially relevant to affect-driven innovative behavior (ADIB) because this form of affect is characterized by elevated activation, goal pursuit, and behavioral readiness. According to AET, some workplace events elicit immediate affective reactions that directly shape subsequent behavior (Weiss & Cropanzano, 1996). When workplace fun is experienced as stimulating, energizing, and socially engaging, it is likely to activate a more action-oriented positive state. Such a state should be particularly relevant to ADIB, which involves the spontaneous generation of ideas and active engagement in novelty. Recent studies likewise suggest that affect-based pathways can promote innovative behavior, although these effects may depend on intervening psychological mechanisms and contextual conditions (He & Li, 2026; Van den Brand et al., 2026).

Workplace fun is an important source of affective reactions and can enhance employees’ positive affect (Fluegge, 2008; Michel et al., 2019). First, workplace fun allows employees to relax during their work and experience pleasure through a series of fun activities (Tang et al., 2017). Employees are more likely to have a positive affect when they genuinely have fun at work. Furthermore, Clark and Watson (1988) discovered that participation in fun activities, particularly social events, could evoke individual positive affect. Workplace fun includes positive activities in which colleagues interact in favorable ways. Therefore, we posit that workplace fun will elevate employees’ positive affect. Additionally, workplace fun fosters a relaxed and pleasant atmosphere and enhances employees’ positive affect, such as satisfaction, joy, and excitement (Shi & Yao, 2019). In light of these observations, we contend that workplace fun could evoke both HMPA and LMPA when employees participate in fun activities.

The increase in HMPA resulting from workplace fun may further promote ADIB, as research shows that participants with positive affect perform better than those with negative affect on creative tasks (Friedman et al., 2007). Positive affect helps broaden thinking, promote team learning, and enhance innovation capability (Kolb & Kolb, 2010). The experiments of Isen et al. (1987) and Isen and Labroo (2003) show that individuals experiencing positive affect tend to think in more flexible and creative ways. Individuals with HMPA may directly engage in innovative behavior, which corresponds to the ADIB described in this study.

Recent research has also begun to move beyond relationship-based explanations and to consider motivational mechanisms through which fun-related practices affect employee outcomes (Han et al., 2024). Among the different forms of workplace fun, sociality-oriented fun may be especially likely to evoke HMPA because it is spontaneous, interactive, and socially energizing. Such activities may generate feelings such as enthusiasm, joy, and activation, which are closely aligned with action-oriented innovative responses. Assistant-oriented fun may also evoke HMPA, although its effects may be less immediate because it is more structured and more closely embedded in work-related activities. Thus, we hypothesize that:

**H2a.**  *HMPA mediates the relationship between sociality-oriented fun and ADIB*.

**H2b.**  *HMPA mediates the relationship between assistant-oriented fun and ADIB*.

### 2.4. The Serial Mediating Role of Low-Motivated Positive Affect and Job Satisfaction

In contrast to high-motivated positive affect (HMPA), low-motivated positive affect (LMPA) is characterized by a calmer, more reflective, and less action-oriented positive state (Gable & Harmon-Jones, 2010, 2016). Such a state is less likely to stimulate immediate behavioral activation but more likely to broaden cognitive appraisal and shape how employees evaluate their work environment. This distinction is important for understanding judgment-driven innovative behavior (JDIB), which, according to AET, emerges not directly from immediate affective arousal, but from affective reactions that first influence work-related judgments and attitudes (Weiss & Cropanzano, 1996).

Job satisfaction is especially relevant in this process because it reflects employees’ evaluative orientation toward their job and work situation. As AET suggests, affective reactions and job satisfaction are conceptually distinct, and affective experiences may serve as antecedents of work attitudes such as job satisfaction (Weiss & Cropanzano, 1996). When workplace fun evokes LMPA, employees may become more likely to interpret their work environment positively, perceive greater support and enjoyment, and develop a higher level of job satisfaction.

This argument is also supported by previous research. Positive affect contributes to psychological resilience and subjective well-being, thereby fostering the development of lasting personal and social resources (Fredrickson, 2001). Employees with positive affect are more likely to feel satisfied with their work (Kafetsios & Zampetakis, 2008). In addition, Staw et al. (1994) found that employees with positive affect tend to receive more favorable evaluations and experience more positive work-related outcomes. In the context of LMPA, employees may be especially likely to evaluate their work situation in a broader and more balanced manner, which may further enhance job satisfaction (Gable & Harmon-Jones, 2010).

Higher job satisfaction may, in turn, encourage JDIB. Employees with more favorable evaluations of their work are more willing to engage in challenging and implementation-oriented activities associated with innovation (Wong et al., 2021). Previous research has also reported a positive relationship between job satisfaction and innovative behavior (Wei et al., 2020; Alshebami, 2021). Therefore, when workplace fun evokes LMPA and subsequently enhances job satisfaction, employees may become more likely to engage in JDIB.

Assistant-oriented fun may be particularly relevant to this pathway because it is embedded in work-related activities and may therefore influence not only employees’ momentary affective experiences but also their broader evaluations of the job context (Y. Wang et al., 2017). Sociality-oriented fun may also contribute to this process, although its effects may be less directly tied to work appraisal. Therefore, we hypothesize that:

**H3a.**  *LMPA and job satisfaction serial mediate the relationship between sociality-oriented fun and JDIB*.

**H3b.**  *LMPA and job satisfaction serial mediate the relationship between assistant-oriented fun and JDIB*.

**Figure 1:**
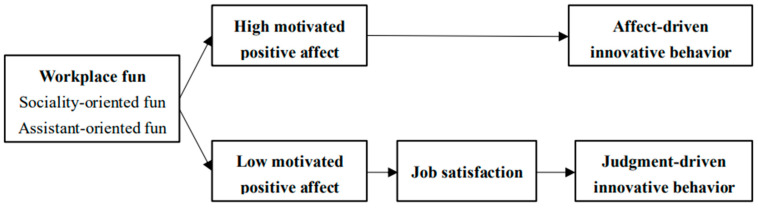
Hypothesized model.

To summarize, hypothesized model of this research is illustrated in Figure 1.

## 3. Research Design

### 3.1. Participants and Procedures

Drawing on existing workplace fun research (e.g., J. Yang et al., 2025), our study was conducted through a questionnaire survey. The survey was administered in a Chinese state-owned electric power company, where workplace fun initiatives are widely practiced. To minimize the effects of common-method variance (CMV), we distributed matched questionnaires to employees and their direct supervisors (Podsakoff et al., 2012). The employee questionnaire included measures of workplace fun, affective states, job satisfaction, and ADIB, whereas direct supervisors evaluated their subordinates’ JDIB. To improve coverage and response rate, we adopted a mixed-mode survey approach that combined paper-based and online questionnaires (De Leeuw et al., 2012). Specifically, 48 paper questionnaires were distributed to local employees through the human resources department, of which 30 were valid. We then distributed online questionnaires to out-of-town employees using an email list provided by the HR department, which yielded 86 valid online responses. In total, we obtained 116 valid responses, representing a response rate of 77.3%. Following the completion of the employee survey, the 116 questionnaires were coded, and new questionnaires were sent to the corresponding 17 direct supervisors for JDIB evaluation. This process resulted in 116 matched survey forms that were usable for further analysis and met the minimum sample size threshold of no fewer than 100 recommended by Everitt (1975).

Of the employee respondents, 69.8% identified as male, and 30.2% as female. Among them, 20.7% belonged to the 22–25 age group, and 79.3% were 26 or older. The majority of participants (62.1%) held a bachelor’s degree, and 37.9% held a master’s degree or higher. Regarding tenure, 30.2% of employees had been at their current workplace for 1–3 years, 35.3% for 4–5 years, and 34.5% for 6 years or more.

### 3.2. Measures

Sociality-oriented fun/Assistant-oriented fun. We used the workplace fun scale developed by Chinese scholars Y. Wang et al. (2017) to measure the frequency of each activity that occurred on a seven-point Likert scale ranging from 1 (strongly inconsistent) to 7 (strongly consistent). Sociality-oriented fun was measured with three items, such as “sharing jokes and stories with coworkers”. Assistant-oriented fun was measured with five items, such as “A small-scale party organized by the team to celebrate good performance”.

HMPA/LMPA. Based on the PANAS scale developed by Watson et al. (1988), three items were screened and extracted to measure HMPA and LMPA respectively. These items were rated on a seven-point scale ranging from 1 (strongly disagree) to 7 (strongly agree). HMPA was measured using items like “active”, “joyful”, and “enthusiastic,” resulting in a Cronbach’s α of 0.94. LMPA was evaluated with items like “interested”, “satisfied”, and “happy”.

Job satisfaction. Employees rated their job satisfaction using three items developed by Cammann et al. (1983) on a seven-point scale ranging from 1 (strongly disagree) to 7 (strongly agree). Sample items included “generally speaking, I like working here”.

ADIB/JDIB. We used a thirteen-item scale adapted from that developed by Kleysen and Street (2001), and modified by Chinese researchers Gu and Peng (2010) for applicability in a Chinese cultural context. This scale was originally designed to measure both idea generation and idea implementation. Direct supervisors rated employees’ JDIB using a six-item, seven-point scale ranging from 1 (strongly inconsistent) to 7 (strongly consistent). Sample items included “He or she once implemented changes that seem to be beneficial”. Employees rated their ADIB using a seven-item scale on a seven-point scale ranging from 1 (strongly inconsistent) to 7 (strongly consistent), with sample items like “I once generated ideas or solutions to address problems at work”. Furthermore, to verify the unidimensionality of the ADIB and JDIB constructs, we conducted confirmatory factor analyses (CFA). The results indicated that both measurement models demonstrated acceptable fit to the data: for ADIB, χ^2^ (11) = 19.74, CFI = 0.99, TLI = 0.97, RMSEA = 0.08, SRMR = 0.03; for JDIB, χ^2^ (6) = 7.95, CFI = 0.99, TLI = 0.98, RMSEA = 0.05, SRMR = 0.03. All fit indices met the recommended criteria.

All items are provided in the Supplementary Materials.

Control variables. Previous studies have shown that employees’ age, gender, and education will impact their innovative behavior (Wallace et al., 2016). Therefore, these variables were considered as control factors in our study.

## 4. Results

### 4.1. Confirmatory Factor Analysis (CFA) and Discriminant Validity Test

Table 1 presents the standardized factor loadings, Cronbach’s α, composite reliability values (CR), and average variance extracted (AVE) of the study factors. All items loaded 0.55 or higher on their respective constructs. CR and AVE values were greater than the recommended 0.70 and 0.50.

We conducted CFAs to examine the discriminant validity of the hypothesized seven-factor model. Given the relatively small sample size (*N* = 116) and the large number of items within the innovative behavior scale (13 items), we employed the item-parceling method as recommended by Little et al. (2013) to reduce model complexity. It should be noted, however, that this approach may obscure item-level variability. The six items of JDIB were randomly parceled into two indicators, and the seven items of ADIB were randomly parceled into three indicators. According to Gorsuch (1974), the sample size should maintain a ratio of at least five participants per measured variable. Following item parceling, our study included a total of 22 measured indicators, thereby meeting this minimum criterion and permitting subsequent statistical analyses.

Results in Table 2 show that the seven-factor model (sociality-oriented fun, assistant-oriented fun, HMPA, LMPA, job satisfaction, ADIB, and JDIB) fitted the data considerably better than alternative models did (Cheung & Rensvold, 2002), χ^2^(188) = 309.69, CFI = 0.93, TLI = 0.92, RMSEA = 0.08, and SRMR = 0.07. Hence, the discriminant validity of the seven-factor model was substantiated.

### 4.2. CMV Test

Given that a substantial portion of our data was collected from employees at a single point in time, there was a potential for common method bias. To address this concern, we conducted Harman’s single-factor test, whereby common method bias is considered a serious concern if factor analysis yields a single factor, if the first factor accounts for the majority of variance (exceeding 50%), or both (Podsakoff et al., 2003). We initially applied Harman’s single-factor test to the data provided by the employees, which included aspects such as workplace fun, affect, job satisfaction, and ADIB. The analysis revealed that the variance explained by the first factor was 39.12%, indicating that common method bias was not a significant issue. Subsequently, we extended Harman’s single-factor test to encompass all data, and the results indicated that the variance explained by the first factor was 34.09%, again falling below the 50% threshold. Lastly, adopting the unmeasured latent method factor approach, we added a latent common method factor into the hypothesized seven-factor model. Although the model’s fit was slightly improved (χ^2^(187) = 301.78, CFI = 0.94, TLI = 0.92, RMSEA = 0.07, and SRMR = 0.08), the changes in these values were not particularly significant as per the criteria set out by Hair et al. (2010). Consequently, common method bias in our study does not appear to be a serious concern.

### 4.3. Correlations and Descriptive Statistics

Table 3 shows the descriptive statistics and correlations for the study variables.

### 4.4. Hypothesis Testing

SEM with Mplus version 8.3 (Muthén & Muthén, 2017) was employed to test our hypotheses. To explore the heterogeneity of workplace fun, we examined the two forms of workplace fun separately. Consistent with the CFA, we parceled the six items of JDIB into two indicators and the seven items of ADIB into three indicators.

We initially examined the direct effects. The results revealed that sociality-oriented fun was positively related to both ADIB (*β* = 0.23, *p* < 0.05) and JDIB (*β* = 0.93, *p* < 0.001). The model fit indices were good (χ^2^(35) = 56.92, CFI = 0.96, TLI = 0.94, RMSEA = 0.07, and SRMR = 0.07). Thus, H1a and H1b were supported. Further, the analysis indicated that assistant-oriented fun was positively related to both ADIB (*β* = 0.58, *p* < 0.001) and JDIB (*β* = 0.19, *p* < 0.05). The model fit indices were again good (χ^2^(56) = 80.42, CFI = 0.95, TLI = 0.93, RMSEA = 0.06, and SRMR = 0.06). Thus, H1c and H1d were supported. More specifically, the effect of sociality-oriented fun on JDIB was statistically significant and relatively strong, suggesting that fun in the form of employee-initiated activities that are less relevant or unrelated to work may be particularly important for JDIB. In contrast, assistant-oriented fun may be more important for ADIB.

**Figure 2:**
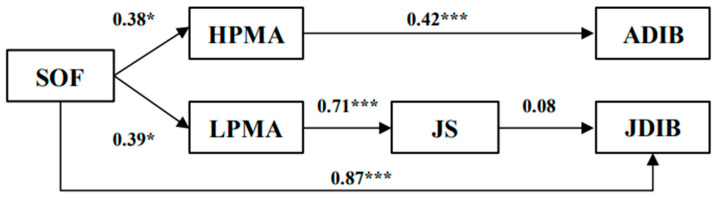
Estimates for model3. * *p* < 0.05; *** *p* < 0.001.

**Figure 3:**
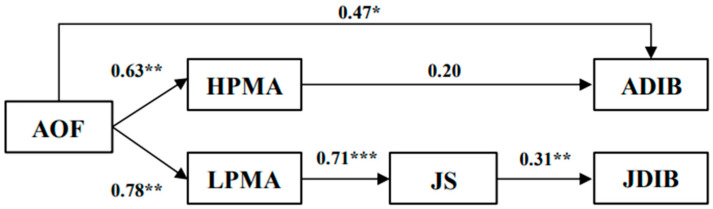
Estimates for model6. * *p* < 0.05; ** *p* < 0.01; *** *p* < 0.001.

Subsequently, we applied the bootstrapping method to test multiple mediated effects, whereby a mediated effect is confirmed only when the indirect effect is significant and the bias-corrected confidence interval does not contain zero (Hayes & Preacher, 2010). To confirm the distinct pathways of the mediated effects of the two forms of workplace fun, we examined eight alternative models encompassing both full and partial indirect effects based on our proposed hypotheses. Models 1 and 5 were fully mediated models without direct paths, featuring sociality-oriented fun and assistant-oriented fun as independent variables, respectively. Models 2 and 6 included an additional path from workplace fun to ADIB, while Models 3 and 7 incorporated another path from workplace fun to JDIB. Models 4 and 8 included two more paths: one from workplace fun to ADIB and another from workplace fun to JDIB. Table 4 displays the fit indices for these eight modes. Model 3 (χ^2^(156) = 220.95, χ^2^/*df* = 1.42, CFI = 0.96, TLI = 0.95, RMSEA = 0.06, and SRMR = 0.08) and Model 6 (χ^2^(195) = 281.75, χ^2^/*df* = 1.44, CFI = 0.94, TLI = 0.94, RMSEA = 0.06, and SRMR = 0.07) showed better fit than the alternative specifications considered. In addition, Models 3 and 4 (and similarly Models 6 and 8) yielded substantively consistent results with only minor differences in parameter estimates. We therefore retained Models 3 and 6 for the mediation and serial mediation analyses because they showed slightly better fit while remaining consistent with the theorized path structure. The path coefficients for models 3 and 6 are depicted in Figure 2 and Figure 3.

The results of the mediation analysis using bootstrapping (as presented in Table 5) demonstrated that sociality-oriented fun significantly influences ADIB through HMPA (*β* = 0.16, *p* < 0.05). This assertion was further supported by the 95% confidence interval (95% CI [0.06,0.33]), which did not contain a zero value. Thus, H2a was supported. However, the indirect effect that assistant-oriented fun on ADIB via HMPA was not significant (*β* = 0.13, *ns*) and the 95% confidence interval (95% CI [−0.01,0.34]) included zero value. Therefore, H2b was not supported.

The results of the serial mediation analysis did not support the indirect association of sociality-oriented fun with JDIB (*β* = 0.02, *ns*, 95% CI [−0.02,0.08], including zero). Consequently, H3a was not supported. Conversely, assistant-oriented fun exhibited significant serial indirect effects on JDIB via LMPA and job satisfaction (*β* = 0.17, *p* < 0.05). In addition, the 95% confidence interval (95% CI [0.06,0.33]) did not contain zero value, thereby supporting Hypothesis H3b.

However, we must acknowledge that although the model fit indices are within acceptable thresholds, the RMSEA value suggests only a moderate fit, indicating that alternative model specifications may also be plausible.

## 5. Discussion and Conclusions

The findings of this study revealed three main results regarding workplace fun (see Table 5): First, both sociality-oriented fun and assistant-oriented fun have positive influences on ADIB and JDIB. Second, HMPA serves as a mediator in the relationship between sociality-oriented fun and ADIB. Finally, the relationship between assistant-oriented fun and JDIB is serially mediated by LMPA and job satisfaction.

### 5.1. Theoretical Implications

This study contributes to the workplace fun literature by examining the impact of specific workplace fun activities on employees’ innovative behavior. Existing studies have often examined workplace fun as a general climate or broad set of positive activities, and have therefore tended to emphasize its overall beneficial effects on employees (e.g., J. Yang et al., 2019; Jing et al., 2021; Lee et al., 2021). In contrast, our findings suggest that different forms of workplace fun may operate in different ways. By distinguishing between sociality-oriented fun and assistant-oriented fun, this study provides a more fine-grained explanation of how fun at work may shape employee innovation. In this sense, this study extends previous research from a general “fun is beneficial” argument to a more differentiated account of when and how specific forms of workplace fun matter. This argument also complements recent studies showing that workplace fun may operate through a broader range of motivational and behavioral mechanisms, such as autonomous motivation, task crafting, and harmonious passion (M. Chen et al., 2025; Han et al., 2024; J. Yang et al., 2024).

Furthermore, this study contributes to the literature on affect by showing that positive affect is not a unitary explanatory mechanism in the relationship between workplace fun and innovative behavior. Drawing on the motivational dimensional model of affect, we distinguish between high-motivated positive affect (HMPA) and low-motivated positive affect (LMPA) and show that these two affective states are associated with different innovative pathways. More specifically, sociality-oriented fun appears to be more closely related to affect-driven innovative behavior (ADIB) through HMPA, whereas assistant-oriented fun is more closely related to judgment-driven innovative behavior (JDIB) through LMPA and job satisfaction. This finding adds theoretical precision to previous studies that have linked workplace fun to employee innovation through broad positive experiences without differentiating the motivational qualities of affect (J. Yang et al., 2019; Michel et al., 2019).

Finally, this study extends the application of AET by integrating its dual-path logic with the motivational dimensional model of affect to explain how workplace fun may influence employee innovation through different mechanisms. While previous studies have mainly focused on the direct pathway, positing that workplace fun incites innovative behavior via affective reactions (e.g., J. Yang et al., 2019), our study supports this view and further examines the indirect pathway, suggesting that workplace fun influences employees’ work attitudes by inducing affective reactions, thereby spurring innovative behavior. Specifically, our findings confirm that only assistant-oriented fun can initiate this sequence of processes. This result indicates that some forms of workplace fun may not primarily operate by immediately energizing employees into action, but by shaping how they feel about and evaluate their work, thereby supporting more reflective and implementation-oriented forms of innovation.

The present findings should also be interpreted in light of the Chinese organizational context, particularly the state-owned enterprise setting in which the data were collected. In Chinese workplaces, employee relationships are often shaped by relatively strong relational expectations, while organizational life is also embedded in clearer formal structures and role boundaries. In a state-owned enterprise, organization-initiated and work-related fun may be especially likely to be interpreted as legitimate and contextually appropriate forms of organizational support, rather than merely as temporary enjoyable activities. By contrast, employee-initiated sociality-oriented fun may function more as a relational and energizing experience among colleagues. This contextual difference may help explain why different forms of workplace fun operate through different affective and attitudinal mechanisms in our model. Accordingly, this study suggests that the effects of workplace fun should not be treated as context-free, and that future research should pay greater attention to how cultural and organizational settings shape employees’ interpretations of fun at work.

At the same time, the present findings should not be interpreted as suggesting that workplace fun is uniformly or unconditionally beneficial. Although both forms of workplace fun examined in this study are associated with positive innovation-related outcomes, it is also plausible that fun activities may become less effective or even counterproductive under certain conditions. For example, excessively frequent, poorly timed, or inauthentic fun activities may distract employees, reduce task focus, or be interpreted as symbolic management rather than genuine support. From this perspective, the value of workplace fun may depend not only on the activity itself, but also on whether employees perceive it as appropriate, meaningful, and compatible with the broader work context. This argument is also consistent with more recent workplace fun research showing that the effects of fun may depend on contextual and individual conditions, such as manager support for fun, employee preference for workplace fun, and evolving work arrangements (Han et al., 2024; Plester & Lloyd, 2024; J. Yang et al., 2024). Together, these considerations point to the importance of examining the boundary conditions of workplace fun in future research.

### 5.2. Managerial Implications

To encourage employees’ innovative behavior, managers should not only promote workplace fun in general but also pay attention to what type of fun is most appropriate for different innovation goals. Our findings suggest that sociality-oriented fun and assistant-oriented fun do not operate in the same way. Sociality-oriented fun is more closely associated with ADIB through HMPA, whereas assistant-oriented fun is more closely related to JDIB through LMPA and job satisfaction. Therefore, managers should promote appropriate forms of workplace fun according to innovation needs within different organizations or departments.

With respect to sociality-oriented fun, managers may facilitate it indirectly by creating conditions that encourage informal interaction and reduce unnecessary isolation among colleagues. In contrast, managers have more direct control over assistant-oriented fun, which can be embedded in work-related activities and designed to strengthen employees’ positive evaluations of their jobs. In this sense, assistant-oriented fun may be particularly useful in settings where innovation depends not only on idea generation, but also on implementation and sustained engagement.

At the same time, managers should ensure that fun activities are consistent with employees’ needs and interests. One practical way to achieve this is to involve employees in determining the forms of workplace fun that are most meaningful and appropriate to them. Moreover, workplace fun should not interfere excessively with routine work schedules or task demands. Its effectiveness is likely to depend on whether employees perceive such activities as authentic, appropriate, and compatible with the work context.

### 5.3. Limitations and Directions for Future Research

This study has several limitations. First, our data were collected from a single large state-owned enterprise in China, which limits the generalizability of our findings to other organizations. At the same time, constrained by the difficulty of collecting matched samples, the final sample size we obtained was not sufficiently large. Future research should adopt large-scale surveys encompassing different types of organizations to explore the impact of workplace fun on employees’ innovative behaviors. Second, our study utilized the AET framework, which is predominantly grounded in a momentary approach. Given the sporadic and frequent occurrence of different fun activities in daily workplace scenarios, capturing the momentary affective states elicited by different types of activities proves challenging. Moreover, research investigating the role of affect in the context of workplace fun remains relatively scarce. Consequently, our study adopted a cross-sectional design, although this is an important limitation. This limitation may also influence the results of our hypothesis testing, particularly the evaluation of the relationship between workplace fun and ADIB. To further probe into the specific pathways between different fun activities, the affects they provoke, and the behavioral outcomes they stimulate, future research should consider adopting a research design that collects daily observational data.

Beyond the methodological limitations noted above, future research would also benefit from adopting a more balanced view of workplace fun itself. In addition, although this study emphasizes the positive role of workplace fun, future research should also examine potential boundary conditions and less beneficial consequences. For example, excessive, poorly timed, or inauthentic fun activities may distract employees, reduce task focus, or be interpreted as managerial control rather than genuine support. A more balanced understanding of workplace fun would therefore benefit from examining both its enabling and constraining effects.

### 5.4. Conclusions

This study aims to clarify how workplace fun can promote employees’ innovative behavior, thus improving our understanding of the relationship between workplace fun and employee innovation. Based on AET and the motivational dimensional model of affect, our findings suggest that the impact of fun activities on employees’ innovative behavior is not uniform but depends on different affective and attitudinal pathways. Specifically, among the two types of workplace fun examined in this study, sociality-oriented fun enhances employees’ ADIB through HMPA. In contrast, assistant-oriented fun increases employees’ LMPA and subsequently improves job satisfaction, thereby contributing to JDIB. These findings suggest that different types of workplace fun may be associated with different forms of innovative behavior through different psychological mechanisms. In this sense, this study contributes to a more fine-grained understanding of how workplace fun may shape employee innovation. At the same time, these findings should be interpreted cautiously in light of the study’s contextual and design limitations, particularly the single-organization setting in a Chinese state-owned enterprise. We therefore encourage future research to employ longitudinal and multi-source designs and to examine broader organizational and cultural contexts in order to further assess the generalizability and boundary conditions of these findings.

## Figures and Tables

**Table 1:** The results of the CFA.

Variables	Items	Standardized Factor Loadings
Sociality-oriented fun Cronbach’s α = 0.77 CR = 0.79, AVE = 0.57	SOF1	0.73
	SOF2	0.92
	SOF3	0.57
Assistant-oriented fun Cronbach’s α = 0.79 CR = 0.86, AVE = 0.56	AOF1	0.72
	AOF2	0.73
	AOF3	0.69
	AOF4	0.76
	AOF5	0.83
HMPA Cronbach’s α = 0.94 CR = 0.92, AVE = 0.80	HMPA1	0.79
	HMPA2	0.98
	HMPA3	0.91
LMPA Cronbach’s α = 0.92 CR = 0.94, AVE = 0.85	LMPA1	0.92
	LMPA2	0.93
	LMPA3	0.91
Job satisfaction Cronbach’s α = 0.77 CR = 0.77, AVE = 0.54	JB1	0.95
	JB2	0.56
	JB3	0.64
ADIB Cronbach’s α = 0.92 CR = 0.93, AVE = 0.64	ADIB1	0.70
	ADIB2	0.76
	ADIB3	0.79
	ADIB4	0.81
	ADIB5	0.87
	ADIB6	0.87
	ADIB7	0.80
JDIB Cronbach’s α = 0.72 CR = 0.89, AVE = 0.58	JDIB1	0.86
	JDIB2	0.87
	JDIB3	0.68
	JDIB4	0.66
	JDIB5	0.83
	JDIB6	0.61

Notes: SOF = sociality-oriented fun; AOF = assistant-oriented fun.

**Table 2:** The results of discriminant validity test.

Measurement Model	χ^2^	*df*	Δχ^2^ (Δ*df*)	CFI	TLI	RMSEA	SRMR
7-Factor model (A; B; C; D; E; F; G)	309.69	188	-	0.93	0.92	0.08	0.07
6-Factor model (A; B; C + D; E; F; G)	359.67	194	6 (49.98)	0.91	0.89	0.09	0.07
5-Factor model (A; B; C + D; E; F + G)	466.54	199	11 (156.85)	0.85	0.83	0.11	0.10
4-Factor model (A + B; C + D; E; F + G)	560.66	203	15 (250.97)	0.80	0.77	0.12	0.11
1-Factor model (A + B + C + D + E + F + G)	921.67	209	21 (611.98)	0.60	0.56	0.17	0.13

Notes: A = sociality-oriented fun, B = assistant-oriented fun, C = HMPA, D = LMPA, E = job satisfaction, F = ADIB, G = JDIB.

**Table 3:** Descriptive statistics and correlations.

Variables	*M*	*SD*	1	2	3	4	5	6	7	8	9
1. Gender	0.30	0.46									
2. Age	2.79	0.41	0.06								
3. Education level	1.38	0.49	0.11	0.36 **							
4. Sociality-oriented fun	5.49	1.15	0.01	−0.12	−0.11						
5. Assistant-oriented fun	4.13	1.36	0.01	−0.04	0.07	0.17					
6. HMPA	3.81	1.61	−0.08	−0.14	0.10	0.26 **	0.46 **				
7. LMPA	3.95	1.66	−0.15	−0.17	0.12	0.23 *	0.50 **	0.85 **			
8. Job satisfaction	4.11	0.84	−0.04	−0.14	0.01	0.16	0.28 **	0.43 **	0.56 **		
9. ADIB	4.65	1.23	−0.21 *	0.02	0.09	0.18	0.49 **	0.44 **	0.44 **	0.44 **	
10. JDIB	4.72	1.04	0.05	−0.08	−0.01	0.56 **	0.23 *	0.30 **	0.30 **	0.27 **	0.34 **

Notes: *N* = 116. For gender: 0 = male, 1 = female. For age: 1 = 21 and below, 2 = 22–25, 3 = 26 and above. For education level: 1 = undergraduate, 2 = postgraduate. * *p* < 0.05; ** *p* < 0.01.

**Table 4:** Summary of model fit indices.

Model Test	χ^2^	*df*	χ^2^/*df*	CFI	TLI	RMSEA	SRMR
Model1	319.75	157	2.04	0.89	0.87	0.10	0.10
Model2	319.18	156	2.05	0.89	0.87	0.10	0.10
Model3	220.95	156	1.42	0.96	0.95	0.06	0.08
Model4	220.49	155	1.42	0.96	0.95	0.06	0.08
Model5	297.79	196	1.56	0.94	0.92	0.07	0.09
Model6	281.75	195	1.44	0.94	0.94	0.06	0.07
Model7	297.78	195	1.53	0.93	0.92	0.07	0.09
Model8	281.24	194	1.45	0.94	0.93	0.06	0.07

**Table 5:** The result of the hypothesis test.

Relationships	Estimate	Standard Error	95% Confidence Interval	Hypotheses	Outcome
sociality-oriented fun → ADIB	0.23 *	0.12		H1a	Supported
sociality-oriented fun → JDIB	0.93 ***	0.11		H1b	Supported
assistant-oriented fun → ADIB	0.58 ***	0.13		H1c	Supported
assistant-oriented fun → JDIB	0.19 *	0.09		H1d	Supported
sociality-oriented fun → HMPA → ADIB	0.16 *	0.07	[0.06,0.33]	H2a	Supported
sociality-oriented fun → LMPA → job satisfaction → JDIB	0.02	0.02	[−0.02,0.08]	H3a	Not supported
sociality-oriented fun → JDIB	0.87 ***	0.19	[0.56,1.29]		
assistant-oriented fun → HMPA → ADIB	0.13	0.08	[−0.01,0.34]	H2b	Not supported
assistant-oriented fun → ADIB	0.47 *	0.21	[0.11,0.97]		
assistant-oriented fun → LMPA → job satisfaction → JDIB	0.17 *	0.07	[0.06,0.33]	H3b	Supported

Notes: *N* = 116. 95% Confidence Interval was obtained from 1000 bootstrap samples. Control variables are not listed. * *p* < 0.05; *** *p* < 0.001.

## Data Availability

The datasets generated during and/or analyzed during the current study are available from the corresponding author upon reasonable request.

## Supplementary Materials

The following supporting information can be downloaded at: https://www.mdpi.com/article/10.3390/bs16050750/s1, File S1: The scales used in this study.
