# Supplementary material for: How Workplace Fun Promotes Employees’ Innovative Behavior: A Dual Mediation Model
Source: Behav Sci (Basel). 2026 May 12;16(5):750. doi: 10.3390/bs16050750 (PMC13203499; doi:10.3390/bs16050750)
Supplement: Supplementary file 1 [file behavsci-16-00750-s001.zip › behavsci-4255551-supplementary.pdf]

File S1. The scales used in this study:

Sociality-oriented fun

1. Coworkers share jokes and light humor with one another.
2. Coworkers get together to discuss trending topics or recent personal experiences.
3. Coworkers share food with one another.

Assistant-oriented fun

1. The organization or department provides refreshments.
2. The team holds small gatherings to celebrate good performance or achievements.
3. The organization provides a pleasant physical environment inside and outside the workplace.
4. The organization improves or establishes leisure facilities in the workplace, such as gyms, basketball courts, badminton courts, and libraries.
5. The organization provides psychological counseling or stress management activities.

Please indicate the extent to which you experienced the following feelings during work today.

High-motivated positive affect

1. Active
2. Joyful
3. Excited

Low-motivated positive affect

1. Amused
2. Content
3. Happy

Job satisfaction

1. In general, I like working here.
2. In general, I do not like my job.
3. Overall, I am satisfied with my position.

Affect-driven innovative behavior

1. I look for opportunities to improve the company, department, work processes, or services.
2. I pay attention to unusual problems in my work, department, company, or market.
3. I generate new ideas or new solutions to problems at work.
4. I look at issues from different perspectives in order to understand them more clearly.
5. I try out new ideas or new methods.
6. Through experimentation, I examine whether new methods have any flaws.
7. I identify the strengths and weaknesses of new methods.

Judgment-driven innovative behavior

1. He or she tries to persuade others of the importance of new ideas or new methods.
2. He or she proactively promotes new methods and creates opportunities for their implementation.
3. He or she takes risks to support new ideas or new methods.
4. He or she engages in changes that may generate beneficial outcomes.
5. When applying new methods to work processes, technologies, products, or services, he or she tries to correct problems caused by those new methods.
6. He or she applies new ideas and methods to daily work to improve work procedures, products, technologies, or services.

Sociality-oriented fun

1. 同事之间分享笑话和小幽默
2. 同事们聚在一起分享热点问题或近期经历
3. 同事之间分享美食

Assistant-oriented fun

1. 单位或部门提供茶点
2. 团队为庆祝取得好成绩的小型聚会
3. 令人愉悦的单位内外部景观环境
4. 完善和新设工作场所的休闲设施（公司健身房、篮球馆、羽毛球馆、图书馆等）
5. 心理咨询或压力调节活动

描述的是您对自己今天工作中的感受:

high-motivated positive affect

1. 活跃的
2. 快乐的
3. 兴高采烈的

low-motivated positive affect

1. 有趣的
2. 满足的

3. 幸福的

job satisfaction

1. 大体上说，我喜欢在这里工作
2. 大体上说，我不喜欢我的职业
3. 总体而言，我满意的我的职位

affect-driven innovative behavior

1. 我会去寻找可以改善公司、部门、工作流程或服务等的机会
2. 我曾经去注意工作中、部门、公司或市场中的不常出现的问题
3. 我曾经在工作上有新的构想或是新的问题解决方法
4. 为了看清楚问题，我会从不同的角度看待事情
5. 我会去尝试新的构想或新的方法
6. 通过尝试，我会检讨新方法是否有错误
7. 我可以发现一些新方法的优缺点

judgment-driven innovative behavior

1. 他(她)曾经尝试说服别人了解新构想或新方式的重要性
2. 他(她)曾经主动去推动新方法并使其有机会被实施
3. 他(她)曾经冒着风险以支持新构想或方法
4. 他(她)曾经从事可能产生好处的改变
5. 当应用新方法在工作流程、技术、产品或服务时，他(她)会设法修正新方法所产生的毛病
6. 他(她)曾经将新的构想和方法应用到日常工作中来改善工作程序、产品、技术或是服务
